# Specialized palliative care for hospitalized patients with SARS-CoV-2 infection: an analysis of the LEOSS registry

**DOI:** 10.1007/s15010-023-02020-z

**Published:** 2023-03-23

**Authors:** Kirsten Schmidt-Hellerau, Claudia Raichle, Maria M. Ruethrich, Jörg J. Vehreschild, Julia Lanznaster, Susana M. Nunes de Miranda, Claudia Bausewein, Maria J. G. T. Vehreschild, Carolin E. M. Koll, Steffen T. Simon, Kerstin Hellwig, Björn-Erik O. Jensen, Norma Jung, LEOSS Study Group, Björn-Erik O. Jensen, Maria Madeleine Ruethrich, Julia Lanznaster, Maria J. G. T. Vehreschild, Kerstin Hellwig, Christoph Spinner, Frank Hanses, Christian Hohmann, Timm Westhoff, Stefan Borgmann, Kai Wille, Jan Rupp, Juergen vom Dahl, Christian Degenhardt, Martin Hower, Christoph Roemmele, Nora Isberner, Lukas Eberwein, Katja Rothfuss, Ingo Voigt, Maria Madeleine Ruethrich, Lorenz Walter, Philipp Markart, Janina Trauth, Secil Deniz, Norma Jung, Gernot Beutel, Milena Milovanovic, Murat Akova, Siri Göpel, Claudia Raichle, Stefani Roeseler, Lars Wojtecki, Mark Neufang, Joerg Schubert

**Affiliations:** 1 Department I of Internal Medicine, University of Cologne, Faculty of Medicine and University Hospital Cologne, Cologne, Germany; 2 Department of Geriatric and Palliative Medicine, Tropenklinik Paul-Lechler Hospital, Tuebingen, Germany; 3 Department of Internal Medicine II, Haematology and Medical Oncology, University Hospital Jena, Jena, Germany; 4 Department II of Internal Medicine, Haematology/Oncology, Goethe University, Frankfurt, Frankfurt Am Main, Germany; 5 German Centre for Infection Research (DZIF), Partner Site Bonn-Cologne, Cologne, Germany; 6 Department II of Internal Medicine, Hospital Passau, Passau, Germany; 7 Department of Palliative Medicine, LMU University Hospital Munich, LMU Munich, Munich, Germany; 8 Department of Internal Medicine II, Infectious Diseases, University Hospital Frankfurt, Goethe University Frankfurt, Frankfurt Am Main, Germany; 9 Department of Palliative Medicine, Faculty of Medicine, University of Cologne, Cologne, Germany; 10 Department of Neurology, St. Josef-Hospital Bochum, Ruhr University Bochum, Bochum, Germany; 11 Department of Gastroenterology, Hepatology and Infectious Diseases, Medical Faculty, University Hospital Duesseldorf, Heinrich Heine University, Düsseldorf, Germany

**Keywords:** Palliative care, Pandemic, COVID-19, Hospitalized patients, Multicentre prospective cohort, Infectious diseases

## Abstract

**Purpose:**

Symptom control for patients who were severely ill or dying from COVID-19 was paramount while resources were strained and infection control measures were in place. We aimed to describe the characteristics of SARS-CoV-2 infected patients who received specialized palliative care (SPC) and the type of SPC provided in a larger cohort.

**Methods:**

From the multi-centre cohort study Lean European Open Survey on SARS-CoV-2 infected patients (LEOSS), data of patients hospitalized with SARS-CoV-2 infection documented between July 2020 and October 2021 were analysed.

**Results:**

273/7292 patients (3.7%) received SPC. Those receiving SPC were older and suffered more often from comorbidities, but 59% presented with an estimated life expectancy > 1 year. Main symptoms were dyspnoea, delirium, and excessive tiredness. 224/273 patients (82%) died during the hospital stay compared to 789/7019 (11%) without SPC. Symptom control was provided most common (223/273; 95%), followed by family and psychological support (50% resp. 43%). Personal contact with friends or relatives before or during the dying phase was more often documented in patients receiving SPC compared to patients without SPC (52% vs. 30%).

**Conclusion:**

In 3.7% of SARS-CoV-2 infected hospitalized patients, the burden of the acute infection triggered palliative care involvement. Besides complex symptom management, SPC professionals also focused on psychosocial and family issues and aimed to enable personal contacts of dying patients with their family. The data underpin the need for further involvement of SPC in SARS-CoV-2 infected patients but also in other severe chronic infectious diseases.

## Introduction

Severe manifestations of SARS-CoV-2 predominantly affect older and frail people as well as those with chronic conditions, leading to hospitalization and life-threatening disease particularly in patients with comorbidities. Managing these patients has placed unforeseen requirements on healthcare infrastructure across all sectors, especially regarding severely ill and dying patients. Globally, 6.5 million people have died from COVID-19 so far, more than 2 million of them in Europe [1]. In Germany, though other countries were affected more severely, the incidence of COVID-19-related deaths was high at 186 per 100,000 inhabitants [1].

In general, palliative and end of life care has become an integral part of care for patients with cancer, and to a lesser degree also for non-cancer patients with other chronic conditions [2, 3]. Involvement of palliative care specialists in infectious diseases is still rather unusual, so far a positive impact of palliative care consultations on antibiotic overuse has been noted [4]. The use of hospital palliative care teams has been shown to have increased during the COVID-19 pandemic [5–7], further demonstrating the need for palliative care in infectious diseases. During the pandemic, effective symptom management and end of life care needed to be provided in the acute care setting in a situation of strained resources and complying with infection prevention measures. This applied not only to intensive care but also to general wards and increased the demand for specialist palliative care (SPC). Early in the pandemic, recommendations for the care of SARS-CoV-2 infected patients from a palliative care perspective have been published, also targeting non palliative care physicians [8–12]. Several studies have been published describing the situations of SARS-CoV-2 infected patients with palliative care needs and what SPC support they received. However, many studies were single centre studies with less than 100 participants [13]. Especially data contextualising SARS-CoV-2 infected patients receiving palliative care support by comparing their case characteristics to SARS-CoV-2 patients not receiving such support is lacking. Therefore, we aimed to describe the characteristics of SARS-CoV-2 infected patients who received SPC and the type of SPC provided, compared to hospitalized SARS-CoV-2 infected patients without SPC support in a larger cohort.

## Methods

### Study design

Data were retrieved from the Lean European Open Survey on SARS-CoV-2 infected patients (LEOSS) registry, a multi-centre non-interventional cohort study. Methods were previously described in detail [14]. For the present study, we analysed data documented between July 2020 and October 2021 of hospitalized patients with confirmed SARS-CoV-2 infection (via PCR diagnosis or rapid tests as an acceptable alternative). Reporting follows the STROBE guidelines [15].

### Participants and setting

The LEOSS data set included both ambulatory and hospitalized patients with SARS-CoV-2 infection at international study centres. For this study, we retrieved data of all inpatients. Patients for which there were missing or unknown data to the question “Did the patient receive specialist palliative care during the SARS-CoV-2 infection?” were excluded from analysis. Hospitalized patients were followed up in LEOSS until end of inpatient treatment or death.

### Data collection and processing

Study sites documented patient data retrospectively and anonymously in an electronic case report form using the online cohort platform ClinicalSurveys.net. To ensure anonymity in all steps of the analysis process, an individual LEOSS Scientific Use File was created, which is based on the LEOSS Public Use File principles described by Jakob et al. [14, 16].

We extracted data on the following characteristics: age, gender, place of stay before SARS-CoV-2 infection and after discharge from hospital, month of diagnosis, symptoms and comorbidities, provision of SPC, personal contacts with family and/or friends before or during the terminal phase, prognosis to live < 1 year, death during hospital stay, type, setting and involved SPC professionals, as well as drugs prescribed for symptom control.

Selected for analysis were symptoms highly prevalent in advanced disease (dyspnoea, delirium, nausea/vomiting, excessive tiredness) and comorbidities leading to limited life expectancy (metastatic tumour disease, dementia, chronic kidney disease on dialysis, liver cirrhosis). Presence of a symptom in at least one phase of the SARS-CoV-2 infection was counted as yes in our analysis.

Binary variables were either documented as yes/no/unknown (comorbidities, drugs, variables regarding type of and profession involved in SPC), or as quoted/not quoted (involvement of palliative care team, symptoms, symptoms unknown, personal contacts). Values documented as unknown were defined as missing in the analysis. Missing data could also be due to blank answer boxes or to adjustments in data collection throughout the project.

### Statistical analysis

Data were analysed using IBM SPSS Statistics 28.01.1. Descriptive analysis included absolute numbers and frequencies of independent categorical variables. Between the groups of patients who received and who did not receive SPC, these frequencies were compared using the Chi Square Test, reported *p* values are two-sided and *p* < 0.05 was considered statistically significant. When applicable, column proportions were compared using the z-test with adjusted p-values (Bonferroni method). Missing data were assumed to be missing at random.

## Results

Data from 7507 patients hospitalized with SARS-CoV-2 infection documented between July 2020 and October 2021 were retrieved from LEOSS. Of these patients, 215 (2.8%) were excluded from analysis because it was unknown if SPC was provided. The observation period (time from the first positive SARS-CoV-2 result to end of inpatient treatment or death) ranged from 1 to 145 days.

**Fig. 1:**
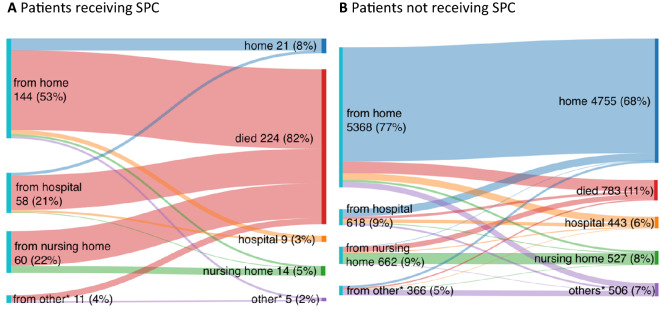
Type of previous residence and residence after discharge of hospitalized SARS-CoV-2 positive patients receiving or not receiving SPC. *Includes discharge to rehabilitation facility or refugee accommodation, homeless persons and others

Of the 7292 patients included in the analysis, 273 (3.7%) had received SPC. Patients receiving SPC were older than patients not receiving SPC and suffered more often from all registered comorbidities (see Table 1 for patient characteristics). Of the patients suffering from metastatic malignancy, 25% (*n* = 48/196) received SPC, as compared to 11% of patients with dementia (*n* = 65/595), 8% of patients with liver cirrhosis (*n* = 6/77) and 7% of patients on dialysis (*n* = 15/221). A large proportion (99/239 (41%)) did not have a disease with an estimated life expectancy < 1 year before admission. Almost half (118/273 (43%) vs 1281/7017 (18%)) were treated in another hospital or a nursing home before this inpatient episode (See Fig. 1).

**Table 1:** Characteristics of hospitalized SARS-CoV-2 positive patients who received and who did not receive SPC

	All patients *n* = 7292	SPC *n* = 273	No SPC *n* = 7019	*p*-Value
	*n* (%)	*n* (%)	*n* (%)	
Gender				
Female	3303 (45)	132 (48)	3171 (45)	0.301
Male	3989 (55)	141 (52)	3848 (55)	
Age^a^				
< 46 y	1463 (20)	5 (2)	1458 (21)	< 0.001*
46–55 y	1004 (14)	17 (6)	987 (14)	
56–65 y	1257 (17)	24 (9)	1233 (18)	
66–75 y	1243 (17)	51 (19)	1192 (17)	
76–85 y	1595 (22)	110 (40)	1485 (21)	
> 85 y	703 (10)	66 (24)	637 (9)	
Pre-existing conditions				
Tumour with metastasis^b^	196 (3)	48 (19)	148 (2)	< 0.001
Dementia^b^	595 (9)	65 (25)	530 (8)	< 0.001
Chronic kidney disease on dialysis^c^	211 (3)	15 (6)	206 (3)	0.014
Liver cirrhosis^d^	77 (1)	6 (2)	71 (1)	0.057

*Statistically significant (< 0.05) for all age groups except “66–75 years”

^a^27 missing

^b^295 missing

^c^282 missing

^d^290 missing

The frequency of the symptoms available for analysis differed between groups. Regarding most symptoms, the group of patients receiving SPC was affected more frequently, as shown in Table 2. Analysing the subgroup of patients with dementia who received SPC (65/260 (25%)), 16/65 (25%) suffered from delirium and 27/65 (42%) from excessive tiredness. Of all patients receiving SPC, 224/273 (82%) died compared to 783/7019 (11%, *p* < 0.001) of those without SPC.

**Table 2:** Selected symptoms of hospitalized SARS-CoV-2 positive patients who received and who did not receive SPC

	All patients *n* = 7292	SPC *n* = 273	No SPC *n* = 7019	*p*-Value
	*n* (%)	*n* (%)	*n* (%)	
Symptoms				
Dyspnoea^a^	3803 (52)	195 (71)	3608 (52)	< 0.001
Delirium^b^	284 (4)	45 (17)	239 (3)	< 0.001
Nausea/emesis^c^	935 (13)	26 (10)	909 (13)	0.123
Excessive tiredness^d^	1447 (20)	84 (31)	1363 (20)	< 0.001

^a^26 missing

^b^64 missing

^c^63 missing

^d^62 missing

Among patients receiving SPC, SPC provided most often symptom control (223/235 (95%)), followed by family support (89/178 (50%)) and psychological support (77/181 (43%)). In almost 9 out of 10 patients (205/237 (86%)), opioids were prescribed for symptom control, followed by benzodiazepines in every 3rd patient (79/210 (38%)). See Table 3 for characteristics of SPC management. Of all patients who died (receiving or not receiving SPC), personal contact with family or friends before or during the dying phase was more often possible in the SPC group (100/194 (52%)) compared to patients without SPC (213/705 (30%), *p* < 0.001). 27/266 (10%) of patients were treated in a palliative care unit.

**Table 3:** Characteristics of SPC management in hospitalized SARS-CoV-2 positive patients (*n* = 273)

	*n* (%)
Which type of specialist palliative care did the patient receive?	
Symptom control	223/235 (95)
Psychological support	77/181 (43)
Social support	64/175 (37)
Spiritual support	35/173 (20)
Family support	89/178 (50)
Which drugs were prescribed for symptom control?	
Opioids	205/237 (86)
Benzodiazepines	79/210 (38)
Neuroleptics	54/206 (26)
Anticholinergics	15/200 (8)
Other	31/192 (16)

## Discussion

Even though end of life care plays an important role in the COVID-19 pandemic and recommendations for the care of critically ill and dying people have been published [8, 17–19], data from this large cohort from a registry study shows that only a small minority of hospitalized SARS-CoV-2 infected patients received specialized palliative care.

Less than 4% of hospitalized SARS-CoV-2 infected patients receiving SPC strongly underpins that the provision of SPC in SARS-CoV-2 infected patients might be insufficient. Of patients included in this study, 14% died during their hospital stay. Therefore, the need for SPC in these acutely ill and acutely symptomatic patients can be assumed to be higher, especially as the value of specialist palliative care consultations, as it has been previously shown, lies not only in clinical management, but also in (re)defining the treatment goal [20].

A previous study looking at critically ill patients in intensive care units has shown a similar proportion of SPC involvement [21], but our data are in contrast to previous data from hospitalized patients that reported a much higher proportion. For example, Golob et al. reported palliative care consultations in New York in 56% of 203 patients who died from COVID-19 [22]. Among the many challenges to provide SPC during the pandemic is the acute care setting, in which the time from acknowledging the need for palliative care to death is shorter than before the pandemic [5, 13]. In a quaternary care centre in New York, the proportion of patients who were supported by palliative care teams who died during the hospital stay increased from 38% to 70% during the pandemic [6]. Among patients supported by palliative care teams, the in-hospital mortality of SARS-CoV-2 infected patients has been shown to be higher than of SARS-CoV-2 negative patients [7, 23]. Another factor potentially contributing to insufficient provision of SPC is that the focus is often on disease rather than symptom management and for many professionals, palliative care is still very much linked to dying and end of life care rather than seeing it as a way of support for symptom management, psychosocial support and advance care planning earlier in the disease trajectory. Furthermore, not all participating centres in our study might have had access to SPC. Involvement is obviously only possible, if there is a SPC service on site (hospital palliative care team). Alternatively, cooperation between hospitals and SPC services would have to be established.

The characteristics of patients receiving SPC differed from patients not receiving SPC regarding comorbidities, symptoms and outcomes. Patients suffering from metastatic malignancy received SPC to a larger proportion than patients with other comorbidities. But when looking at the group of all patients receiving SPC, dementia was a more frequent diagnose than metastatic malignancy due to the high rates of dementia among patients hospitalized with SARS-CoV-2. This is of clinical relevance for future SPC planning, because patients with dementia require special considerations, for example higher rates of delirium have been shown in this study confirming previous data (see below) [24]. More generally speaking, it has been previously described that during the pandemic the pre-existing conditions of patients supported by palliative care services shifted towards less patients with end stage organ disease and cancer and more patients with obesity and diabetes [7]. In the present cohort, patients receiving SPC were older and suffered more often from comorbidities limiting life expectancy. At the same time, a large proportion (almost 60%) of patients receiving SPC did not have an underlying disease with an estimated life expectancy of less than one year, which means that in many cases therapeutic goals were shifted during the hospital stay in the course of the acute infectious disease. This is in line with data from the United Kingdom, showing that 87% of SARS-CoV-2 infected patients receiving palliative care services had not been supported by palliative care before their infection [25]. Kamal et al. described that 69% of hospitalized SARS-CoV-2 infected patients where full code before involvement of palliative care, but only 28% afterwards [20]. This shows that, especially in a pandemic situation, SPC teams provide support in defining the individual goal of care and that advance care planning has a great potential impact.

During their hospital stays, patients receiving SPC more frequently suffered from dyspnoea, delirium and excessive tiredness than patients not receiving SPC. The most common symptom, affecting over two-thirds of patients in this cohort, was dyspnoea, which is in line with previous data from SARS-CoV-2 infected patients referred to palliative care [26] and from SARS-CoV-2 infected patients during their last week of life [27]. The proportion of dyspnoea in SARS-CoV-2 infected patients receiving palliative care and during the last week of life seems to be slightly higher compared to patients suffering from other diseases [27–29].

Delirium was a frequent symptom in patients receiving SPC, observed in 17%. High rates of delirium have been described previously in SARS-CoV-2 infected patients referred to hospital palliative care (24%) [5, 26], and even higher in SARS-CoV-2 infected patients referred to hospital care coming from a nursing home (41%) [28]. Alderman et al. have observed delirium in 56% of SARS-CoV-2 infected patients who died later [29]. Data from a facility for patients with dementia also suggested that in these patients, delirium is frequently among the first symptoms of COVID-19 (37%) [30]. In the present study, patients with dementia are a large subgroup of patients receiving SPC (25%) who need special attention. Apart from increased rates of delirium (25%), patients with dementia also more frequently suffered from extensive tiredness (almost half of them). Along these lines, Swedish registry data from a large cohort showed that of hospitalized SARS-CoV-2 infected patients, 7% suffered from dementia, and that of these 57% developed a delirium [27], which was observed more often during the last week of life than in SARS-CoV-2 negative patients [27]. In this registry data, 82% of SARS-CoV-2 infected patients receiving SPC did not survive their hospital stay. This is in line with previous data (74%) [26].

Patients receiving SPC more often had personal contact with family before or during the dying phase (52%). Without SPC, this was true for only 30%, similar to what has been described in the above-mentioned Swedish registry study (26%) [27]. Even though SPC seems to support personal contact in these situations, there is still room for improvement regarding the importance for patients and families.

The distress for both patients and clinicians created by visiting restrictions has been previously described [31]. A systematic review concluded that visiting restrictions led to increased pain, loneliness, depressive symptoms, agitation, aggression and reduced cognitive ability in patients, as well as to anxiety in family members [32]. Lonely dying of patients has been described as a major family concern [33], and it is assumed that it negatively impacts on bereavement [34]. Supporting patients and relatives during visiting restrictions is, together with working under infection prevention precautions and integration of palliative care in other clinical settings, one of three major aspects identified by a scoping review of palliative care in pandemic settings by Gesell et al., and it has been strongly recommended to enable visits in some way as this is of great importance to the patient and families [34].

One of the main limitations of this study is the registry methodology. The data does not allow to draw any robust conclusions to what extent and why patients might not have received SPC even though it would have been indicated (due to resource constraints, local lack of expertise, not included in therapeutic planning because other specialties are less used to these types of patients were involved). The study design does not allow for comparison of SPC provided for SARS-CoV-2 positive patients to e.g. cancer patients in general. Furthermore, pain as a highly prevalent symptom in patients with advanced disease has not been included in the registry and could thus not be analysed. However, the open registry also holds a number of advantages, such as collection of data of a high number of patients on important topics under constrained circumstances.

## Conclusion

During the current pandemic, some hospitalized SARS-CoV-2 infected patients already presented with pre-existing life limiting comorbidities, but in many the course of the viral infection led to a change of the therapeutic goal to palliative symptom management and end of life care. The value of SPC has been previously shown both for patient management and for defining treatment goals. Considering that treatment goals were changed in many cases and that in-hospital mortality of patients hospitalized with SARS-CoV-2 infection was high, current low rates of involvement of SPC in these patients can be assumed to be insufficient.

Not only dyspnoea, but also other symptoms like delirium, for which high rates have been shown especially among patients with dementia, required complex palliative symptom management, thus posing an additional and new challenge for professionals during the pandemic. Particularly that one quarter of the patients hospitalized with SARS-CoV-2 infection receiving SPC suffered from dementia has practical implications for clinical care and SPC planning.

Another challenge in the setting of infection control was to enable personal contact with family before or during the dying phase, which was more often provided to patients receiving SPC.

## Data Availability

The data of this study can be requested from the corresponding author in justified cases. The access to the LEOSS scientific data (Scientific Use File, or SUF) needs to be discussed within and confirmed by the LEOSS Board of Investigators. A LEOSS public dataset (Public Use File, or PUF), with a corresponding dashboard, is available on the LEOSS homepage (https://leoss.net/data/).
